# Geographic accessibility to public dental practices in the Jazan region of Saudi Arabia

**DOI:** 10.1186/s12903-022-02279-y

**Published:** 2022-06-22

**Authors:** Mosa Ali Shubayr, Estie Kruger, Marc Tennant

**Affiliations:** 1 International Research Collaborative, Oral Health and Equity, School of Human Sciences, The University of Western Australia, Nedlands, Australia; 2 Department of Preventive Dental Sciences, College of Dentistry, Jazan University, Jazan, Kingdom of Saudi Arabia; 3 School of Human Sciences, Faculty of Science, The University of Western Australia, 5 Stirling Highway, Crawley, WA 6009 Australia

**Keywords:** Accessibility, Dental healthcare services, Health facilities, Geographic information systems, Jazan, Saudi Arabia

## Abstract

**Background:**

It is impossible to attain good general health without maintaining oral health and this becomes problematic when dental services are located far from the population that needs to utilise them. This study aimed to assess the geographic accessibility of dental clinics located in public primary healthcare clinics (PHCs) and hospitals in the Jazan region of Saudi Arabia and how long it takes to reach them by car and on foot.

**Methods:**

The location of clinics and hospitals, maps of road systems, and the governorates' borders (administrative areas) within the Jazan region were downloaded using the QGIS mapping tool. The time taken to travel to the clinics and hospitals, either by driving or walking, was assessed. If the time was 30 min or less, residents in the area were classified as ‘serviced’. It was more than 30 min, they were ‘underserved’.

**Results:**

Only 31% of Jazan residents were found to live in a serviced area if they drove to clinics and hospitals. Residents of Jazan's seven mountainous governorates were more likely to require services. Only 40% could drive less than 30 min to a primary health dental clinic. Only 19% of people could walk to a hospital in less than 30 min. Only two governorates had a majority of residents who lived in serviced areas.

**Conclusion:**

The study demonstrates an accessibility issue, as many Jazan inhabitants must drive or walk for an extended period (> 30 min) to reach a healthcare facility, whether a primary health care centre or a hospital. This issue may result in many people not receiving necessary health care, compromising their oral health status. Additional research is needed to identify public, private and other health facilities in the region and the prevalence of oral disease.

## Introduction

Everyone has a fundamental need for health. However, good general health is difficult to attain without maintaining good oral health because the oral cavity is regarded as a mirror of the body [1]. This becomes more challenging when dental services are placed in areas remote from the population that needs them. Even a modest increase in the distance between healthcare facilities decreases health service consumption, such as complete dental examinations [2]. A systematic review found that lack of transportation was one of the things that kept people from getting dental care [3].

Access means ‘that degree of fit between the system and the patient’ [4]. Different dimensions help provide an overview of access to healthcare. Accessibility, availability, accommodation, affordability, and acceptability are examples of access dimensions [4]. Accessibility is determined by the relationship between the location of health services and the location of a patient. The World Health Organization [5] defines accessibility in various ways, including economic, informational, and physical [5]. Each level of accessibility addresses a unique set of health demands and obstacles. Access to pertinent information reaffirms an individual's or group's right to seek and receive information about a health condition. Once informed of their need, there is still a financial factor to consider as well as the proximity of the desired health services to individuals in need.

Seriousness, kernel density, and catchment area methodologies have all been viable ways to measure health accessibility [6]. However, the Euclidean and network distance methods continue to be the most frequently used in public health research [7]. The Euclidean distance approaches are primarily motivated by the relationship of an area to a point or locations of supply based on the crow-flies distance. The network distance is the route travelled to reach the destination [8].

There is no universally acknowledged distance allowed for people to travel for medical care. This means that no standard criteria can be used to measure how far is far enough for healthcare services' accessibility. However, stakeholders like the WHO advise using journey time rather than travel distance to measure the accessibility of healthcare services. This approach evaluates the state of the roads and means of transportation [9].

According to some authors, a half-hour should be allotted for patient care [10]. Others argue that people who live more than three-quarters of an hour away from healthcare facilities are more likely to be marginalised [11]. Others consider one hour to be adequate [12].

Numerous research has been conducted in Saudi Arabia on the subject, although most of it has concentrated on the barriers to dental care utilisation. The location of dental clinics was a significant factor affecting dental care usage [13]. According to one study conducted in southern Saudi Arabia, 17% of participants could not get care due to the absence of a clinic near their school or home [14]. Another study in which parents completed the questionnaire discovered that the most significant barrier was health literacy (82% of the participants indicated this), while roughly 23% mentioned financial difficulties, transportation difficulties, and lengthy waiting periods [15]. Additionally, rural areas had lower dental care utilisation than metropolitan areas [16], and this was deemed a risk factor for poor oral health [13].

**Fig. 1:**
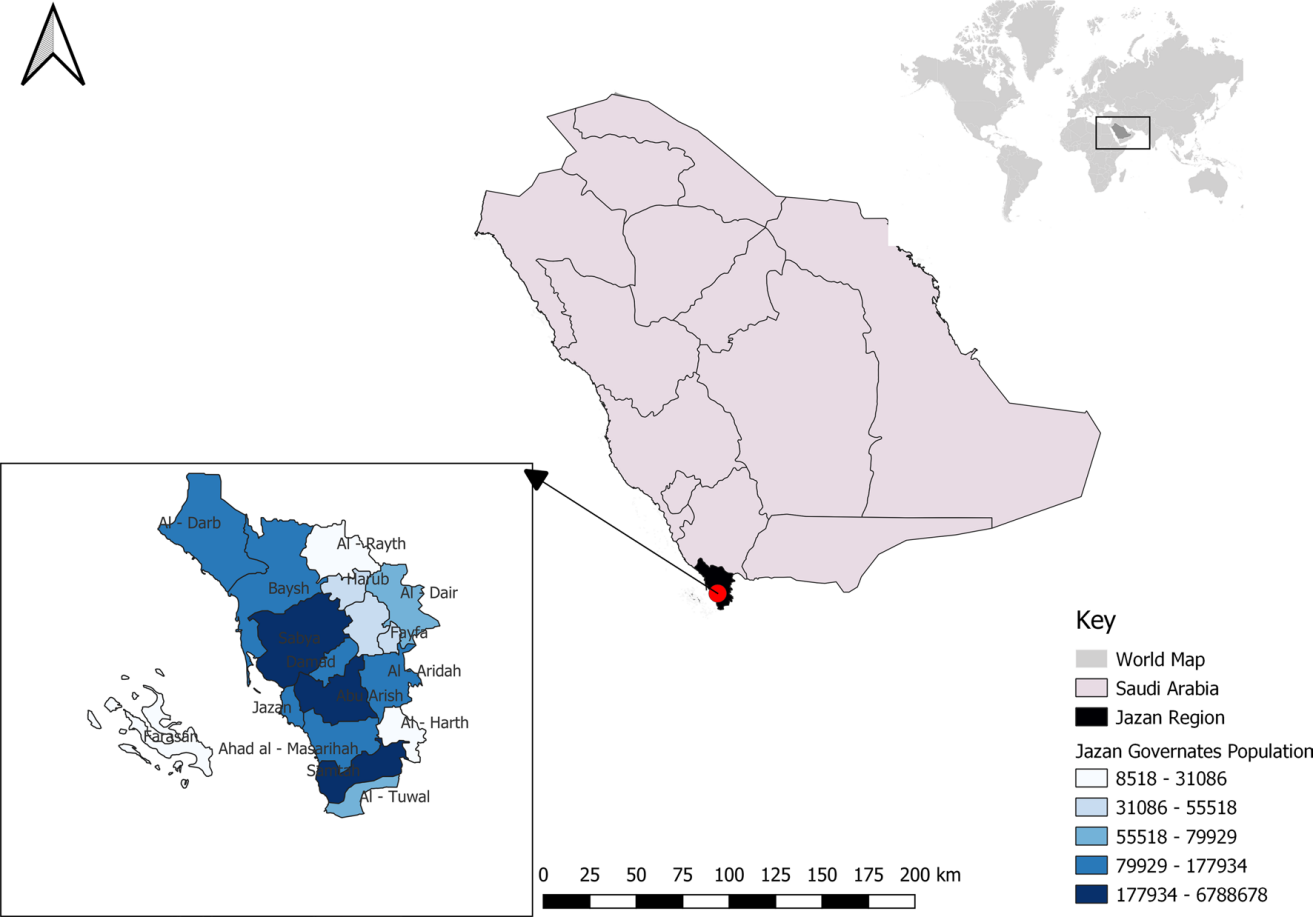
Jazan region map

This study examined travel times to primary health care centres and hospitals with a dental element in the Jazan region (Fig. 1). Jazan is in the southwestern region of Saudi Arabia. This region (also spelt Jizan, Gizan, or Gazan) consists of 17 governorates (*mohafadat* in Arabic). It has a population of approximately 1.7 million—54% male and 46% female [17]. Jazan is rapidly developing as a large agricultural heartland and provides many economic opportunities for domestic and foreign investments. This continued growth has led to healthcare challenges such as increased population, resulting in high health care costs. Jazan is considered one of the regions with the lowest healthcare quality in the country [18].

Proximity to health centres is hypothesised to promote good health by increasing healthcare access [19]. On the other hand, longer travel times and distances obstruct access to healthcare services and discourage repeat visits [20]. This is supported by a comprehensive review, which revealed that around 77% of the 108 studies showed an association between good health outcomes and population location relative to healthcare facilities [21].

According to the authors’ knowledge, there are no comparable published studies of distance to health care services in the same region (Jazan). This study investigates the travel time to dental health care centres and services provided by Ministry of Health (MOH) facilities in the Jazan region. By assessing the region's geographical accessibility, recommendations for future oral health care services can be made. This will provide policymakers with a legal basis to establish more oral healthcare facilities in underserved areas. Furthermore, the findings will be a beacon for further investigation into this domain.

## Material and methods

This study calculated the travel time to the healthcare services and the healthcare facilities by driving or walking scenarios. The study's focus was on primary health care and hospitals with a dental setup in the Jazan region of Saudi Arabia. Data for the current study were obtained between Oct 1st, 2021 to Dec 31st, 2021. The researcher used freely available, anonymous data. Therefore, ethics approval was not necessary.

Each Jazan governate was given its own identifier and its borders were obtained from census data [17, 22]. The road system was obtained from the same source and was divided into two types—main roads and highways. Administrative governates were picked as the geographical region because this was the only available information source in the file format that matched the population information files. All population data were acquired from the most recent data on the General Authority and World Population's websites [23, 24].

The locations of health facilities were determined using the Ministry of Health's Statistical Yearbook [25] and an interactive map created by the Ministry of Health [26]. This study included only MOH primary healthcare facilities and hospitals with dental components, totalling 145 PHCs and 19 hospitals. Other establishments were omitted, including dental schools, mobile clinics, private medical centres, and repeated addresses. Google Maps was used to translate the locations of the health institutions to longitude and latitude, with 90% at the building level.

The researchers carried out spatial mapping in the current study using Quantum Geographic Information Systems (QGIS) (version 3.20, Essen, Germany). The World Geodetic System 1984 was utilised to coordinate referencing in this investigation (WGS 84). Microsoft Excel gathered and analysed geographical and associated population data (version 14.0; Microsoft, Redmond, WA, USA).

This study used two scenarios to measure the travel time—both by driving and on foot. This study did not measure travel time with the straight-line distance method because this does not consider physical barriers such as expanses of water, rail tracks, buildings and other obstacles [8, 27]. Thus, this study measures driving and walking time in minutes using the road system. The travel time isochrones for walking and driving were created using the QGIS python plugin. The Travel Time QGIS plugin incorporates aspects of a distance-journey-time matrix to assist in calculating travel time. The fields for estimated time in minutes in both forward and backward directions were created using a default speed of 5 kms per hour for walking and 80 kms per hour for driving a car [8]. The maximum travel time considered for a serviced area was 30 min. Areas located more than 30 min away from health facilities were considered underserved.

Vehicle movement was identified by road network mapping and spatial data modelling [8]. The researcher discovered during the data cleansing process that certain health institutions were located too far from road networks, potentially confounding the results. The researcher updated the road network by digitising selected road segments from Google Earth [28]. These were then exported to the QGIS spatial analysis software.

This study’s data were analysed both descriptively and by calculation. Data were reviewed using Microsoft Excel (2016 version 14.0). Study data to be analysed were imported from the integrated database in QGIS into Microsoft Excel software.

## Results

**Fig. 2:**
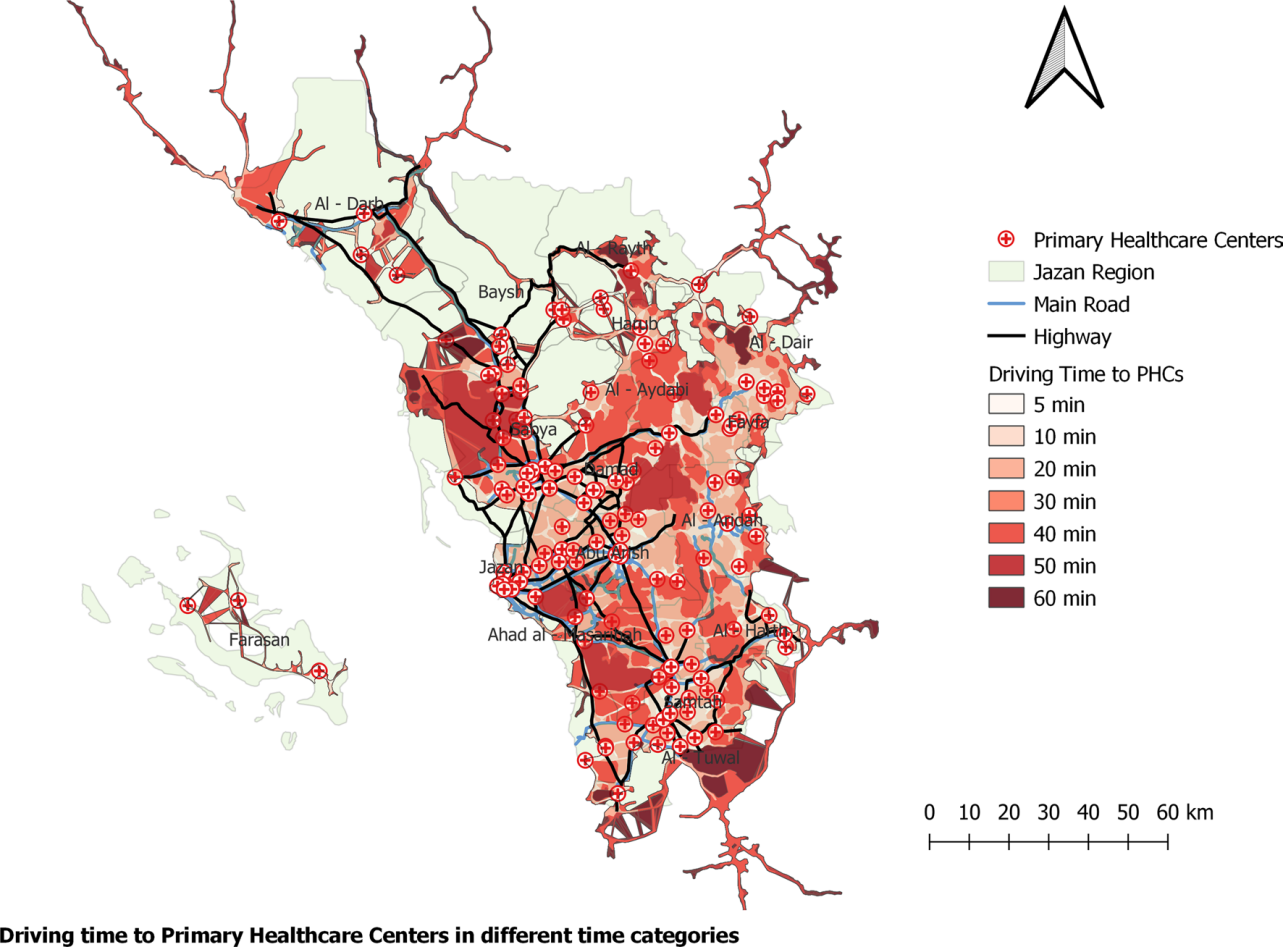
Driving time to primary healthcare canters in different time categories

**Fig. 3:**
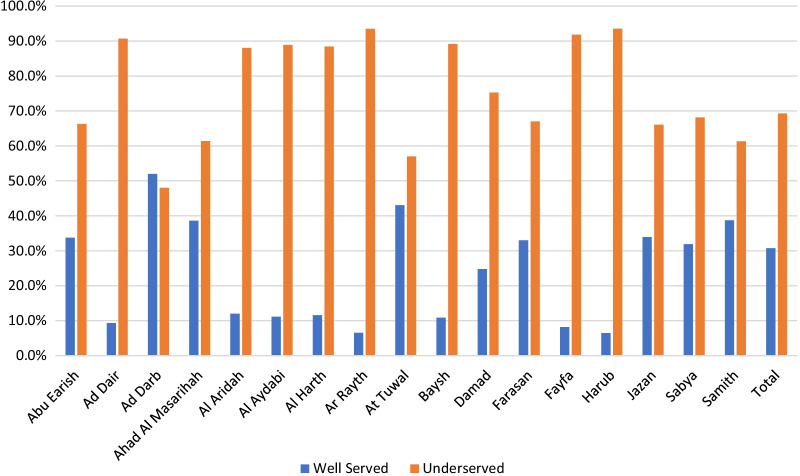
Population in areas served and underserved by primary healthcare centres in the driving times in Jazan cities, Saudi Arabia

The study shows the driving time to 145 primary healthcare centres (PHCs) with a dental component in 7 time categories—5, 10, 20, 30, 40, 50 and 60 min that were geocoded by QGIS (Fig. 2). The findings show that most of the region's residents need a long time to reach the PHC facilities. Only Al Darb and Al Tuwal have a short travel time to PHCs. At 30 min, these are the best-served areas in the region. However, residents in Al Dair, Al Aridah, Al Rayth, Fayfa and Harub have the longest travel time to the PHCs since most of them require 60 min to reach the medical facilities. The results show that the majority (69%) of Jazan region residents live in underserved areas (Table 1). Al Darb, At Tuwal, Jazan and Samtah are the governorates with the highest number of people served by PHCs. (Fig. 3). Most of the mountain governorates such as Al Aridah, Al Aydabi, Al Harth, Ar Rayth, Baysh, Fayfa and Harub had the highest number of underserved populations.

**Table 1:** Summary of the population distribution in the two scenarios

	Driving		Walking	
	PHCs	Hospitals	PHCs	Hospitals
Population Served (≤ 30 mn)	530,566 (30.73%)	552,019 (97%)	690,696 (39.69%)	328,080 (18.72%)
Underserved Population (> 30 mn)	1,196,173 (69.27%)	1,174,720 (68.03%)	1,036,043 (60.31%)	1,398,659 (81.82%)
Total	1,726,739 (100%)	1,726,739 (100%)	1,726,739 (100%)	1,726,739 (100%)

**Fig. 4:**
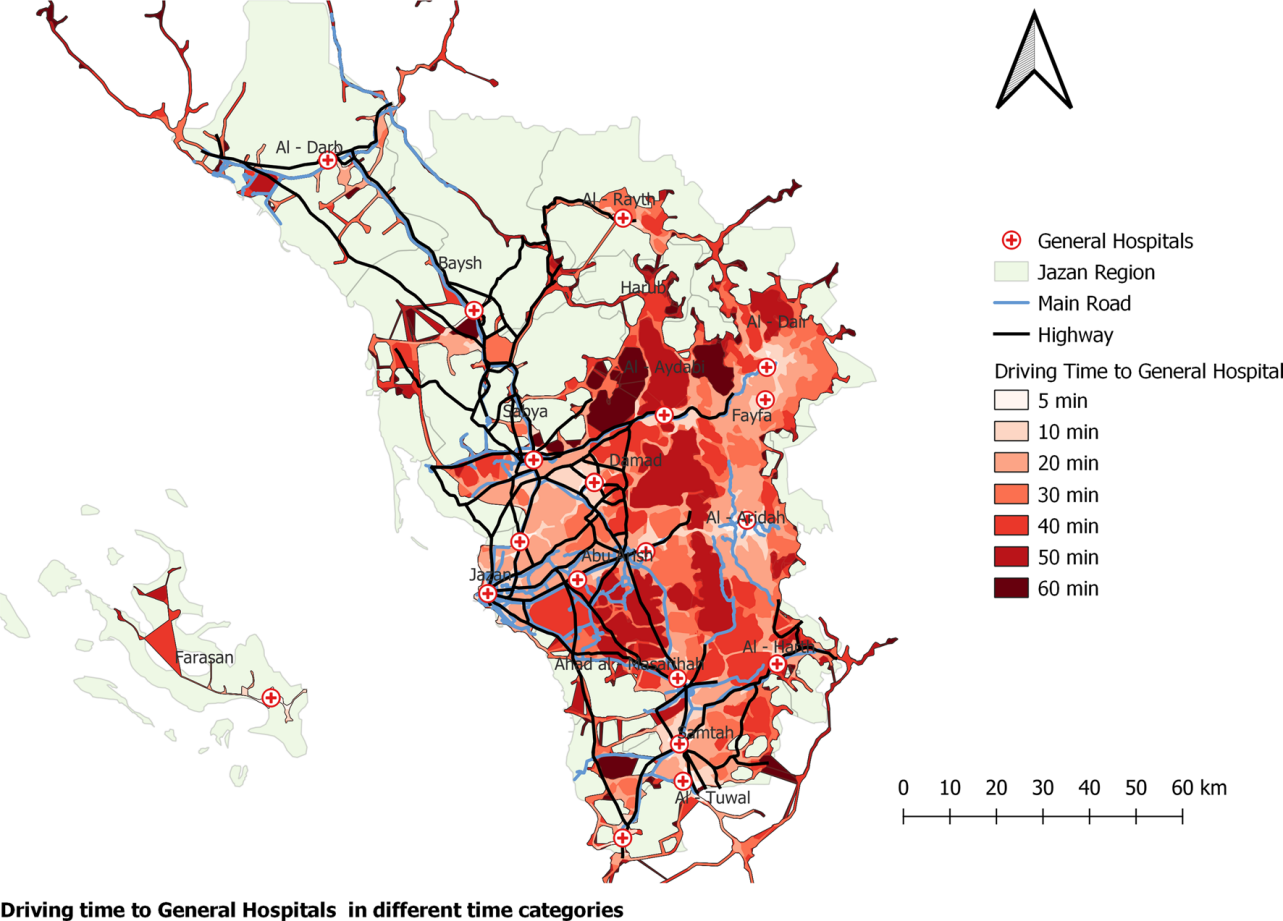
Driving time to general hospitals in different time categories

**Fig. 5:**
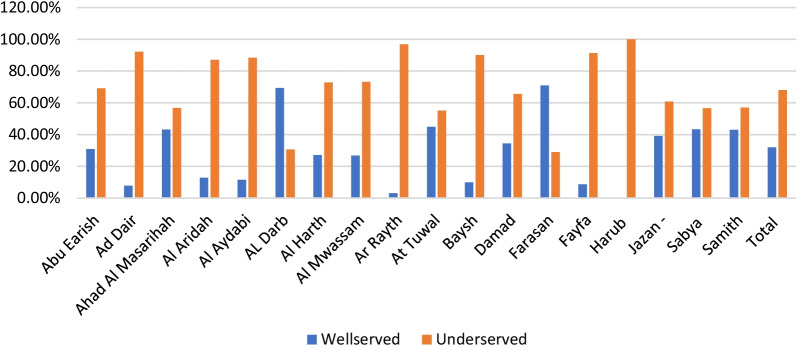
Population in areas served and underserved by hospitals in the driving times in Jazan cities, Saudi Arabia

Regarding driving time to MOH hospitals with the dental component, the findings show that most Jazan region residents have a long driving time to receive health care from hospitals in the region (Table 1 and Fig. 4). Farasan and Al Darb have the shortest travel times to hospitals, at 5 and 20 min. These are, therefore, the best-served areas in the region. Residents of Al Dair, Al Aydabi, Baysh and Fayfa have the longest travel time to the hospitals since most of them need 60 min to reach the services. The findings show that the majority (68%) of Jazan region residents live in underserved areas (Table 1). Al Darb and Farasan are the governorates with the highest number of people living in the areas served by hospitals (Fig. 5). Ad Dair, Al Aridah, Al Aydabi, Al Harth, Ar Rayth, Baysh and Fayfa have the highest number of underserved people and no general hospital in the Harub governorate.

**Fig. 6:**
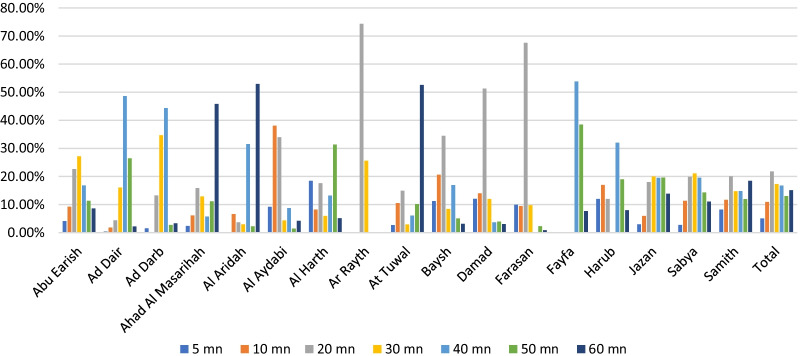
Distribution of walking times to primary healthcare centres in Jazan region cities, Saudi Arabia

**Fig. 7:**
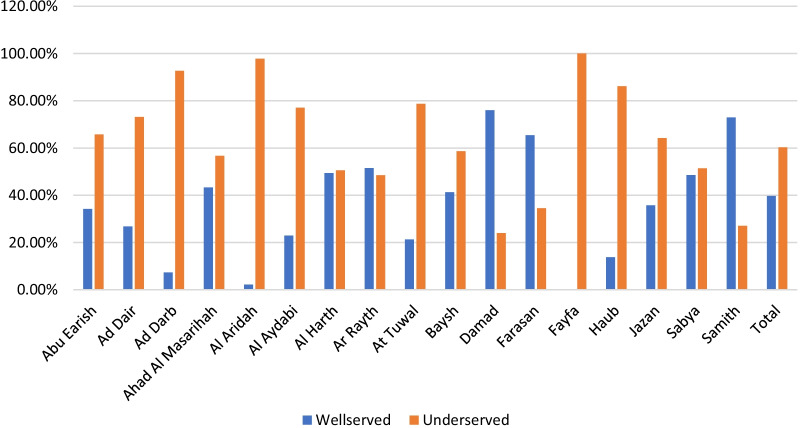
Population in areas served and underserved by primary healthcare centres in the walking times in Jazan cities, Saudi Arabia

The exact time break in the driving scenario was used (Fig. 6). The study found that about 40% of Jazan region residents need 30 min or less to reach the services in PHCs (Table 1). Most residents who live in Ar Rayth, Damad, Farasan and Samitah can reach a PHC service if they walk 30 min or less. Fayfa, Al Aridah and Harub residents can reach PHCs only if they walk for longer than 60 min (Fig. 7).

**Fig. 8:**
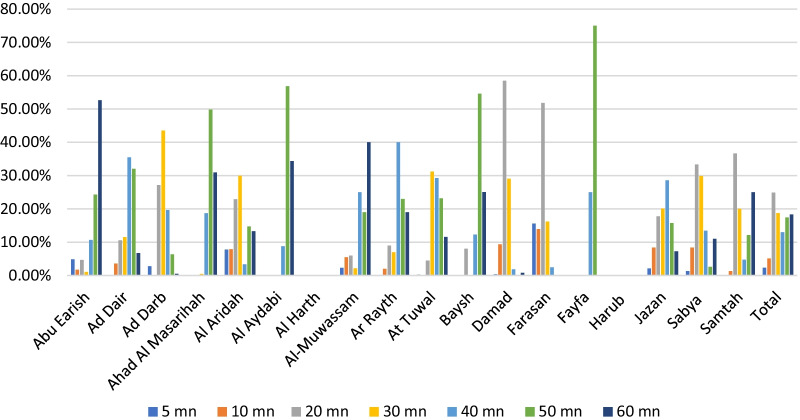
Distribution of walking times to general hospitals in Jazan region’s cities, Saudi Arabia

**Fig. 9:**
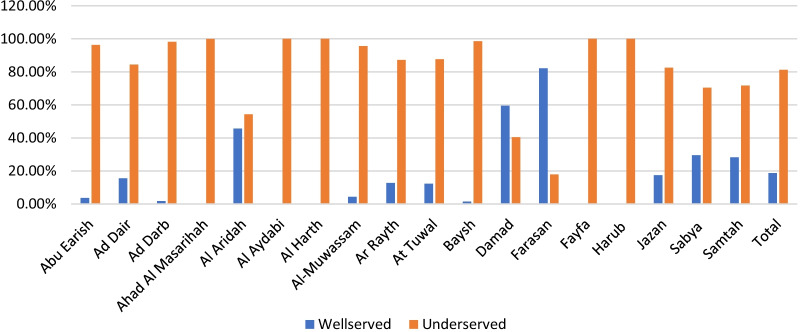
Population in areas served and underserved by hospitals in the walking times in Jazan cities, Saudi Arabia

Regarding walking to the hospital, the researcher used the same time break in the driving scenario (Fig. 8). The study found that about 19% of Jazan region residents need 30 min or less to reach the services in general hospitals (Table 1). The results show in Fig. 9 that more than half of Farasan and Damad residents need to walk for 20 min to reach general hospitals since 82% and 60% of their populations live in areas served by hospitals. Residents of Al Aydabi, Al Harth, Fayfa and Harub need a longer time to reach the health service at hospitals since these areas have the highest number of underserved populations and no general hospital in the Harub governorate.

## Discussion

The current study characterised the diversity in healthcare services in Saudi Arabia's Jazan region through the use of pragmatic estimates of travel time by driving and walking to healthcare centres with dental treatment facilities.

More than any other metric, travel time accurately represents accessibility to healthcare centres. This study corroborated previous research showing that the maximum travel time to a health service should be between 5 and 60 min [8, 29], with served areas taking less than or equal to 30 min and underserved areas taking more than 30 min [30].

This study’s outcomes indicate a problem in the Jazan region concerning timely access to dental health services. The current study’s results are consistent with the findings of previous investigations. In Jeddah, Saudi Arabia, in 2018, it was discovered that several areas of the city lack access to health centres due to their location more than 30 min away [30], and in Makkah, Saudi Arabia, in 2021, it was discovered that a third of the city's population lacks adequate access to health care services due to the majority of health facilities being concentrated in the city's urban area, while rural and remote areas were neglected [31].

These findings are consistent with a 2016 study conducted in Mozambique, which discovered that many people require extensive travel time to access health care [8, 31]. This finding contradicted a study conducted in France, where 75% of the population needed less than 25 min to obtain health treatment [32].

Many residents of Jazan must drive for an extended period (> 30 min) to reach a health facility, whether it is a primary health care centre or a hospital. Residents of Al Darb have a shorter travel time to health services than residents of other governorates. This is explained by the equal population distribution, with half of the population living within the served area, reducing travel time to oral health care services. Additionally, Al Darb is located at the gateway to the Jazan region, allowing residents of Al Darb to easily travel to other closed regions for dental care. Additionally, the study discovered that most mountain governorates, including Al Aridah, Al Aydabi, Al Harth, Ar Rayth, Baysh, Fayfa, and Harub, had the largest proportion of individuals who lacked access to basic services. This could be because these places have fewer roads and more challenging driving conditions. This result is consistent with a previous study, which reported disparity in health care services due to geographical variance [33].

In the walking scenario, only 40% of Jazan region inhabitants require less than 30 min to reach PHCs, and only 19% require less than 30 min to reach hospitals, which is consistent with the study that discovered inadequate accessibility in the walking situation [34]. This could be explained by the absence of sidewalk infrastructure in this area, resulting in restricted access to facilities, which is consistent with the findings of the previous study [35]. However, the bulk of the population of Farasan and Damad was regarded to be in areas served by PHCs and hospitals. This could be because many individuals reside close to the government centres, and the area has an excellent infrastructure for walking.

In terms of population distribution, the study discovered that accessibility is more challenging for residents who need to walk to general hospitals; more than 80% of the Jazan region is underserved. The accessibility issue is almost identical to driving to primary health care centres (69%) or hospitals (68%). This analysis postulated that the entire population possessed a car, widely regarded as the primary mode of transport in Saudi Arabia [29]. This could be due to the small size of the population of these states, the fact that their basic infrastructure is still being developed, or the lengthy driving time to the facility [36]. Additionally, this could be because the majority of the region's residents live in major cities such as Sabya, Jazan, and Abu Arish, which is consistent with a study conducted in Makkah, which found that the bulk of the population lives in the city and one-third of the population lives far from health facilities, implying that they lack adequate access to health care services [31]. Also, a study in Jeddah discovered that many peripheral areas lacked health centres [29].

To address the consequences of insufficient dental service distribution, policymakers should consider expanding the number of health care facilities, particularly in underserved areas. Additionally, the public transportation network's infrastructure must be improved by constructing new roads or the rehabilitation of existing ones. Additional effort is required to map travel times to MOH, private, and other healthcare facilities throughout the region. Additional spatial and attribute variables regarding oral health status, dental services, providers, and the locations of private and other health facilities should be included. Additionally, a qualitative study will ascertain the public's and dental providers' concerns about dental health care access in Saudi Arabia.

To the researcher's knowledge, this is the first study to use QGIS to describe travel time to public dental healthcare services in Saudi Arabia's Jazan region. However, several limitations to the study should be considered. To begin with, there is a dearth of current information about dental health facilities (PHCs and hospitals). This study relied entirely on publicly available data and geographic divisions. At the time of the study, data on oral health services and providers were unavailable at the district level in the Jazan region, including the number of oral health providers (dentists, dental hygienists, and assistants), the type of service offered, waiting time, and characteristics of dental providers such as gender and speciality. Due to the rapid growth of the oral health and general health care systems, the data used in this study may not accurately reflect contemporary advancements. Additionally, the study excluded additional variables such as the location of patients seeking treatment, public transportation, and demographics such as age and gender from the calculation of realistic walking travel time. These issues should be looked into more intensely in future studies to get more accurate estimates of trip times.

Despite these shortcomings, this study possesses many strengths. This study estimated travel time to a health centre using the road network rather than straight lines. The road travel line is more precise and is frequently utilised by the general population [8, 37]. Additionally, the study used updated population data at the state level rather than at the administrative level to avoid skewed or aggregated population data. This study is especially beneficial for informing the distribution of health care facilities and the supply and inequalities in service provision in the region. Furthermore, this study will aid academic and health planners in determining journey times and road networks while also taking the mode of mobility into account.

## Conclusion

This study employed two distinct scenarios to determine the journey time between locations throughout the Jazan region and the nearest healthcare facility, and it provides new information about the region's accessibility to healthcare facilities with a dental component. The study demonstrates that there is an accessibility issue because many Jazan inhabitants need to drive or walk for more than 30 min to reach a healthcare institution, whether it is a primary health care centre or a hospital. This issue may result in many people not receiving necessary health care, affecting their oral health state and ultimately, their overall health.

## Data Availability

All population datasets analysed during the current study are available on the General Authority [https://www.stats.gov.sa/en/1007-0] and World Population's [https://www.worldpop.org/] websites. The location of health facilities was obtained from the Ministry of Health Statistical Yearbook [https://www.moh.gov.sa/en/Ministry/Statistics/book/Pages/default.aspx] and a Ministry of Health interactive map [https://www.moh.gov.sa/en/eServices/interactive-maps/Pages/default.aspx#/].
